# Influence of Cell Detachment on the Respiration Rate of Tumor and Endothelial Cells

**DOI:** 10.1371/journal.pone.0053324

**Published:** 2013-01-30

**Authors:** Pierre Danhier, Tamara Copetti, Géraldine De Preter, Philippe Leveque, Olivier Feron, Bénédicte F. Jordan, Pierre Sonveaux, Bernard Gallez

**Affiliations:** 1 Louvain Drug Research Institute, Biomedical Magnetic Resonance Research Group, Université catholique de Louvain (UCL), Brussels, Belgium; 2 Institut de Recherche Expérimentale et Clinique (IREC), Université catholique de Louvain (UCL), Brussels, Belgium; University of Bari & Consorzio Mario Negri Sud, Italy

## Abstract

Cell detachment is a procedure routinely performed in cell culture and a necessary step in many biochemical assays including the determination of oxygen consumption rates (OCR) *in vitro*. *In vivo*, cell detachment has been shown to exert profound metabolic influences notably in cancer but also in other pathologies, such as retinal detachment for example. In the present study, we developed and validated a new technique combining electron paramagnetic resonance (EPR) oximetry and the use of cytodex 1 and collagen-coated cytodex 3 dextran microbeads, which allowed the unprecedented comparison of the OCR of adherent and detached cells with high sensitivity. Hence, we demonstrated that both B16F10 melanoma cells and human umbilical vein endothelial cells (HUVEC) experience strong OCR decrease upon trypsin or collagenase treatments. The reduction of cell oxygen consumption was more pronounced with a trypsin compared to a collagenase treatment. Cells remaining in suspension also encounter a marked intracellular ATP depletion and an increase in the lactate production/glucose uptake ratio. These findings highlight the important influence exerted by cell adhesion/detachment on cell respiration, which can be probed with the unprecedented experimental assay that was developed and validated in this study.

## Introduction

The oxygen consumption rate (OCR) of cells is an important biological parameter studied in various fields as an indicator of mitochondrial activity. In tumor biology, cancer metabolism studies and OCR measurements have been decisive to show that a decrease in mitochondrial respiration and enhanced aerobic glycolysis (known as the Warburg effect) are linked with tumor aggressiveness [1]–[3], and that endothelial cells submitted to hypoxia undergo profound metabolic changes [4]. OCR is also widely assessed in tumor treatment response and was instrumental to demonstrate that a blockade of mitochondrial respiration, supporting an increase in the local pO_2_, is a valid combinatory treatment option to improve chemo- and radiosensitivity [5]–[9]. Concerning other pathologies, oxygen metabolism is investigated in retinal detachment where restoring appropriate oxygenation can prevent vision loss [10], [11]. More generally, OCR is also an accepted viability marker used for tissue transplantation [12].

**Figure 1:**
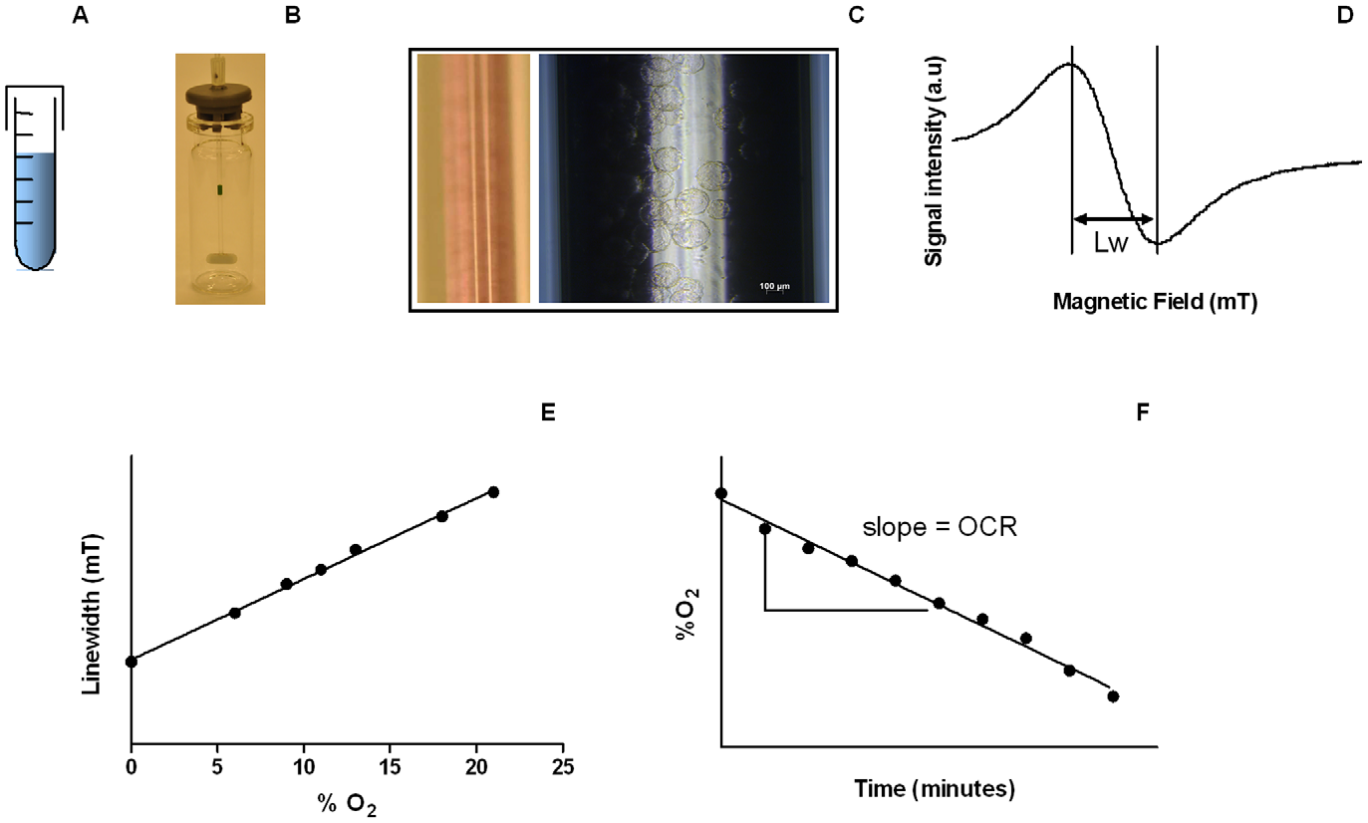
Experimental setup. (A) Cells were incubated with cytodex microbeads during 4 hours at 37°C. (B) Cell-coated microcarriers were placed in a thermostatized (37°C) minispinner in a medium containing 10% (w/v) dextran. (C) Cell-covered microbeads were mixed with a nitroxide (^15^N-PDT) oxygen probe and drawn into a capillary tube which was then sealed, this last one was inserted in the EPR X-band cavity for measurements (D) EPR spectra of ^15^N-PDT were recorded, the linewidth (Lw) being proportional to %O_2_ (E). (F) Recorded linewidths allowed determining the evolution of %O_2_ inside the closed capillary containing adherent cells. The slope is actually the oxygen consumption rate (OCR).

Several techniques are currently used for measuring OCR *in vitro*. The most common ones rely on the use of polarographic Clark electrodes, phosphorescent or fluorescent probes, or electron paramagnetic resonance (EPR) [13], [14], whereas other methods (based on spectrophotometry or near-infrared spectroscopy for example) have been described but are less used [15], [16]. It has been recently demonstrated that EPR is much more sensitive than fluorescence assays or the use of Clark electrodes [13], which is directly attributable to the physics of EPR oximetry as extensively described in recent comprehensive reviews [17], [18]. Briefly, OCR determination using EPR oximetry is based on the signal variation of a paramagnetic oxygen sensing probe in contact with cells sealed in a glass capillary tube (Figure 1). In this setting, cells consume O_2_ at a rate mainly dictated by their mitochondrial activity.

The present study is based on previous works having demonstrated *in vivo* that cell detachment promotes tumorigenesis and leads to metabolic alterations reflected by decreased glucose uptake and decreased ATP levels [19]. A similar metabolic reprogramming could therefore impose a signal drift on the most commonly used OCR assays that generally involve cell harvesting for technical reasons [20]–[22]. Hence, characterizing the impact of cell adhesion and detachment on OCR might also lead to a better understanding of cell-cell and cell-surface interactions, which necessarily involves the design and validation of an appropriate OCR measurement technology. To reach that goal, we aimed to set up and validate a new protocol combining the high sensitivity of EPR and the use of cytodex microcarriers in order to study the influence of cell adhesion and detachment on OCR *in vitro*. Cytodex 1 and collagen-coated cytodex 3 carriers consist in 100-µm diameter dextran microbeads on which cells can easily adhere and grow [23]. Adherent cells can then be handled similarly to cells in suspension, implying that, once carefully validated with microbeads, EPR oximetry could be amenable for the OCR measurement of intact adherent cells.

## Materials and Methods

### Layout of the General Setup

The general objective of the experimental approach is to measure the OCR of two intact, adherent cell lines, namely tumor and endothelial cells, which represent two major cell populations in tumors. The impact of cell detachment on OCR will be studied using two modalities used in classical metabolomics, i.e. the use of trypsin or collagenase. The general strategy is displayed in Figure 1. Cells were coated onto cytodex microcarriers. On the next day, cells on microbeads were poured into a homemade minispinner (adapted from [24]) in a dextran-rich medium at 37°C to avoid sedimentation. Cells were then carefully aspirated into an eppendorf cup (treated with trypsin or collagenase for the detached group), mixed with an EPR oxygen-sensing nitroxide probe, and drawn into a glass capillary tube for EPR measurements. Trypsin or collagenase treatment modalities are summarized in Table 1.

**Table 1:** Summary of the different experimental conditions.

Cell line	Group	Detachment procedure
B16F10-luc	Control	Microbeads covered by cells in full medium with dextran 10% (w/v) (final volume: 300 µl)
B16F10-luc	Trypsin	0.05% Trypsin-EDTA 10 minutes at 37°C followed by addition of full medium with dextran 20% (w/v) (final volume: 300 µl)
B16F10-luc	Collagenase	Collagenase (10 mg/ml) 15 minutes at 37°C (final volume: 300 µl)
HUVEC	Control	Microbeads covered by cells in full medium with dextran 10% (final volume: 100 µl)
HUVEC	Trypsin	0.05% Trypsin-EDTA diluted 2.5 times 5 minutes at 37°C followed by addition of full medium with dextran 20% (w/v) (final volume: 100 µl)
HUVEC	Collagenase	Collagenase (10 mg/ml) 15 minutes at 37°C with dextran 20% (w/v) (final volume: 100 µl)

### Cell Culture

Murine melanoma B16F10-luc cells constitutively expressing luciferase (Xenogen) were grown in Dulbecco′s Modified Eagle Medium (containing GlutaMAX™ I, 1000 mg/L *D*-glucose, sodium pyruvate), supplemented with 10% fetal bovine serum, and 1% (v/v) penicillin/streptomycin (Invitrogen). Human umbilical vein endothelial cells (HUVECs, Sigma-Aldrich) were grown in the Endothelial Cell Growth Medium of Sigma-Aldrich.

### Cell Loading onto Cytodex Microbeads

On the day prior to the experiment, confluent cells were rinsed with PBS, harvested using 0.05% Trypsin-EDTA (Invitrogen), and admixed with full medium (1∶1). After centrifugation (180 g, 5 min), the cells were resuspended in full medium containing sterilized Cytodex 1 (30 mg for B16F10-luc) or collagen-coated Cytodex 3 dextran (30 mg for B16F10-luc, 65 mg for HUVEC) microbeads (Sigma-Aldrich) in a 17*100 mm round bottom tube (BD Biosciences). The tube was then placed during 4 hours in an incubator at 37°C and flicked every twenty minutes during incubation to allow uniform anchorage and distribution of the cells onto the microbeads. After incubation, cell-coated microbeads were placed overnight in a T75 flask (Sarstedt) with full medium at 37°C/5%CO_2_.

### Adherent Cell Preparation for EPR Oximetry

Cells on dextran microbeads were collected and placed into a round bottom tube during 3 minutes in order to settle. After medium removal, the cell-coated beads were washed gently with 10 ml of PBS. A 100 µl aliquot of the suspension was collected, diluted 10 times with PBS in a microtube, and three 10 µl aliquots of this suspension were used to determine the bead concentration under an inverted microscope. Another 100 µl sample was then collected from the 10 ml suspension, mixed with 360 µl 0.05% Trypsin-EDTA and a 40 µl dextranase solution (Sigma-Aldrich) for dissolving the beads (15 minutes at 37°C with continuous agitation) [25]. Released cells were then counted using a hemocytometer. The PBS supernatant of the stock suspension was discarded and replaced by 2 ml of full medium containing 10% (w/v) dextran (molecular weight: 64000–76000) (Sigma-Aldrich). This final suspension was carefully transferred into a thermostatized (37°C) homemade minispinner flask with 40 rpm agitation as already reported [24]. This procedure allows the cells to remain intact and adherent for several hours. Each experiment was performed with cells coming from the same minispinner, implying that cell concentration was exactly the same for control (adherent) and treated (trypsin or collagenase) groups. For oxygen measurements with adherent B16F10-luc cells, 100 µl of cell-coated microbeads were mixed with 190 µl of full medium containing 10% (w/v) dextran and 10 µl of 2 mM oxygen-sensing probe ^15^N-4-oxo-2,2,6,6-tetramethylpiperidine-d_16_-^15^N-1-oxyl (^15^N-PDT). For adherent HUVECs, 95 µl of the microbead solution were mixed with 5 µl of ^15^N-PDT. An equal volume of each cell preparation was then drawn into a capillary tube, the tube was sealed and introduced in a Bruker EMX EPR spectrometer operating at 9 GHz. EPR spectra were recorded every other minute for linewidth determination. All samples were kept at 37°C during all measurements.

### EPR Calibration

The linewidth of^ 15^N-PDT was calibrated at various %O_2_ using different mixtures of air and nitrogen with an Aalborg gas mixer. Exact %O_2_ was defined using a Servomex oxygen analyzer OA450. Ten microliters of ^15^N-PDT 2 mM was mixed with medium containing 20% (w/v) dextran and PBS (ratio 1∶1) for the control group, or microbeads (0.5 mg cytodex 1/50 µl PBS) for the cytodex 1 group.

### Trypsinization

For the trypsinized B16F10-luc group, 100 µl of microbeads were rinsed twice with PBS in a microtube and trypsinized with 200 µl (final volume) of 0.05% Trypsin-EDTA for 10 minutes at 37°C with gentle agitation. After cell harvesting, trypsin was neutralized with 90 µl of full medium containing 20% (w/v) dextran and 10 µl of ^15^N-PDT was added. This solution was then transferred into a capillary tube for EPR measurements. HUVEC trypsinization was performed as follows: 95 µl of HUVEC-coated microbeads were collected and washed twice with PBS in order to reach a final volume of 45 µl. Thirty microliters of 0.05% Trypsin-EDTA were added during 5 minutes at 37°C. Trypsin was then neutralized with 20 µl of full medium containing 20% (w/v) dextran, and 5 µl of ^15^N-PDT was added prior to EPR measurements.

### Collagenase Treatment

One hundred microliters of B16F10-luc grown on cytodex 3 were rinsed twice with PBS, treated with 90 µl of a collagenase type 1 solution (10 mg/ml) (Worthington) during 15 minutes at 37°C with occasional agitation. One hundred microliters of full medium containing 20% (w/v) dextran and 10 µl of ^15^N-PDT were subsequently added, and the resulting cell suspension was drawn into a capillary tube for EPR measurements. The procedure used for HUVEC collagenase treatment was the same as that for HUVEC trypsinization except that trypsin was replaced by a collagenase type 1 solution (10 mg/ml) and the cells were incubated during 15 minutes at 37°C.

### Cell Death Assay

Cell death after trypsin or collagenase treatment was determined using the trypan blue method. Briefly, treated cells were mixed with a trypan blue solution (Sigma-Aldrich, ratio 10∶1). Detached cells and remaining adherent cells on the microbeads were spread onto a 6-well plate. Pictures were acquired using a Zeiss Axiovert S100, from which dead and living cells were counted using the ImageJ software.

### Mitochondrial Loss Following Cell Detachment

Potential mitochondrial loss during cell harvesting from cytodex microbeads using 0.05% Trypsin-EDTA and collagenase was determined with the detection of mouse mitochondrial complex IV subunit 1 (COXI) by western blotting. Briefly, aliquots of 100 µl of B16F10-luc cells loaded onto cytodex 3 microbeads were rinsed twice with PBS and resuspended in 0.05% Trypsin-EDTA or in a collagenase solution (10 mg/ml) for 10 minutes with gentle agitation. After cell detachement, PBS was added and samples were centrifuged (100 g for 5 minutes). Supernatants were discarded and cells were washed with PBS. After a last centrifugation step, samples were resuspended in Laemmli Buffer 2× (6,25% (v/v) Tris HCl 1M pH 6.8–6.9, 10% (v/v) glycerol, 2% SDS, 5% (v/v) β-mercaptoethanol, 0.02% bromophenol blue to match a total volume of 200 µl). As control, cell-coated microbeads were washed several times with PBS and resuspended in 200 µl Laemli Buffer 2×. After denaturation (3 minutes at 100°C), 30 µl of the samples were loaded in triplicate in a 10% Mini-PROTEAN TGX Precast Gel (Bio-Rad). After electrophoresis, proteins were transferred onto a 0.4 µm PVDF membrane using a Trans-Blot Turbo transfer system (Bio-Rad). The membrane was then probed with a mouse anti-COXI antibody (Invitrogen, 1/1000) in PBS containing 0.1% Tween 20 and 5% non fat dry milk (PBST/5% milk) for 2 hours at room temperature with continuous agitation. After two washes with PBST, a peroxidase-conjugated anti-mouse secondary antibody (Jackson ImmunoResearch) was added (1/10000). Chemiluminescense was visualized using Western lightning plus ECL (PerkinElmer). Bands were quantified using the ImageJ software. β-actin was used as a loading control and probed using a mouse anti-β-actin antibody (Sigma-Aldrich, 1/10000).

### ATP Quantification Assay

B16F10-luc cells were grown in 100-mm diameter dishes until they reached confluency. Adherent cells were washed with PBS and recieved full medium for 1 hour at 37°C/5%CO_2_. Then, cells were washed with PBS and collected in 3 ml of cold lysis buffer (10 mM Tris, 1 mM EDTA, 100 mM NaCl, 0.01% Triton X-100). For the detached groups (trypsin and collagenase), confluent cells were rinsed with PBS, detached using 0.05% Trypsin-EDTA (5 minutes at 37°C/5%CO_2_) and neutralized with full medium. At this step, cells were counted using a hemocytometer and viability was assessed with the trypan blue dye. B16F10-luc cells were centrifuged (1000 rpm for 5 minutes) and resuspended in full medium. Detached cells were kept in suspension in an open 15 ml tube in full medium at 37°C/5% CO_2_ with gentle agitation. After 1 hour in suspension, cells were centrifuged, counted and resuspended in 3 ml of cold lysis buffer. Intracellular ATP levels were then detemined using a bioluminescence-based ATP Determination Kit (Invitrogen) following manufacturer’s instructions. Bioluminecence was measured using a SpectraMax M2 microplate reader (Molecular Devices). These experiments were repeated using collagenase instead of trypsin. The same protocol was applied except that B16F10-luc were seeded on BD BioCoat™ collagen I culture dishes (VWR) and cells were harvested using a collagenase type 1 treatment (10–15 minutes at 37°C).

### Glucose Uptake and Lactate Production Assays

B16F10-luc cells were grown in 100-mm diameter dishes (trypsin treatment) or collagen 1-coated dishes (collagenase treatment) until they reached confluency. Cells were washed with PBS, detached using trypsin (5 minutes at 37°C) or collagenase (10–15 minutes at 37°C with continuous agitation). After neutralization with full medium, cells were centrifuged (1000 rpm, 5 minutes) and resuspended in 2 ml of full medium. Cells were kept in suspension for 3 hours (collagenase) or 4 hours (trypsin) at 37°C/5% CO_2_ with gentle agitation. After incubation, viability was assessed using the trypan blue dye and supernatants were collected. As a control, confluent dishes were rinsed with PBS and replenished with 2 ml of full medium. After 3 hours (collagenase experiment) or 4 hours (trypsin experiment) at 37°C/5%CO_2_, supernatants were also collected. All supernatants were next deproteinizided using ultra-centrifugation filter tubes with a 10 kDa cutoff (VWR). Glucose and lactate concentrations were measured using a CMA600 enzymatic analyzer (Aurora Borealis) according to manufacturer’s instructions. Deproteinized full medium was used to precisely determine starting basal glucose and lactate concentrations.

### Statistical Analyses

All results are represented as means ± standard error of the mean (SEM). For the ^15^N PDT calibration curves, linear regressions and comparisons between slopes (Student’s *t*-test) were performed. Student’s t-test was used for OCR, glucose, lactate and ATP comparisons between adherent and detached cells. COXI expression was analyzed using one way ANOVA. *p*<0.05 was considered to be statistically significant.

## Results

### Dextran Microbeads do not Affect Oxygen-sensing by ^15^N-PDT in EPR Oximetry

**Figure 2:**
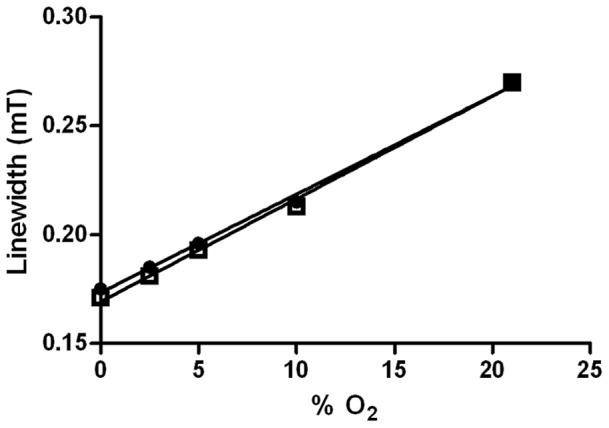
Calibration of ^15^N-PDT in function of %O_2_. Linear regression of nitroxide alone (•) does not differ from calibration of ^15^N-PDT with cytodex 1 beads (□).

In a first instance and in order to validate the subsequent experiments, we tested whether naked cytodex microbeads introduced a bias in recorded %O_2_ values with EPR or not. Calibration curves of ^15^N-PDT obtained with and without dextran microbeads were not statistically different (Figure 2), providing a demonstration that the use of cytodex microbeads is compatible with EPR OCR assays.

### Cell Detachment Induces a Significant Decrease in the Respiration Rate of Tumor and Endothelial Cells

Our validated EPR setup was exploited to question the importance of cell adhesion/detachment on the respiration rate of tumor and endothelial cells, i.e. two cell types representing major cell subtypes found in tumors. In each case, cell detachment was performed using two different treatments, trypsin or collagenase, in order to assess the general effect of detachment but also specificities inherent to the harvesting method.

**Figure 3:**
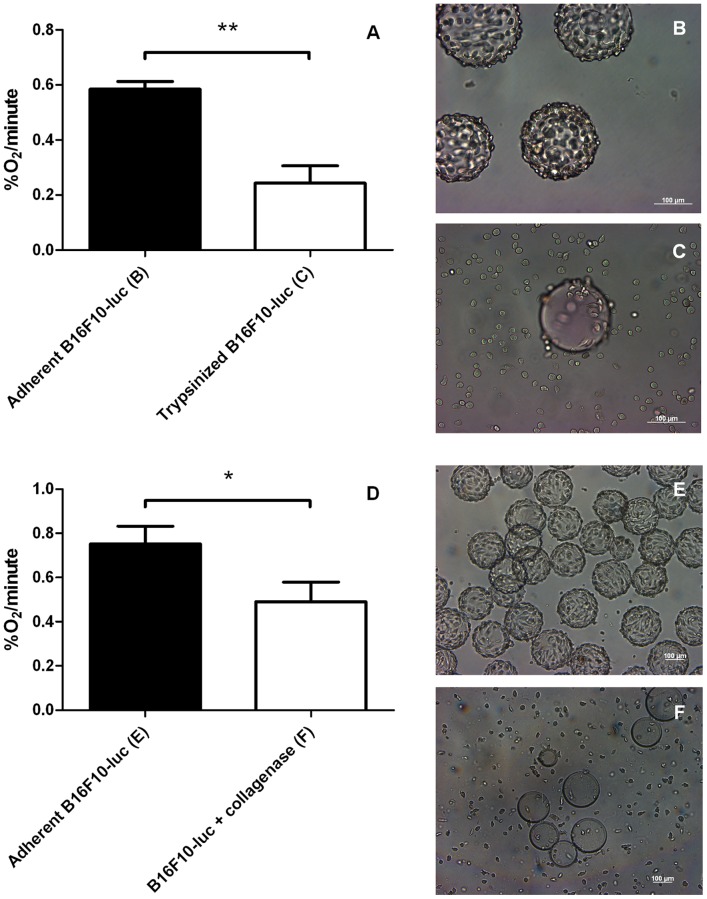
Effect of detachment procedures on B16F10-luc tumor cells. OCR values (%O_2_/minute) (A, D) of adherent B16F10-luc and detached B16F10-luc. Trypsinized (n = 3) or collagenase group (n = 4) show a decreased oxygen consumption rate compared to control groups (n = 3 for A, n = 6 for D). Results are statistically significant (**p<0.01 for trypsinized cells, *p<0.05 for B16F10-luc+collagenase). Pictures of each treatment are displayed next to the graphs. Trypsin treatment (C) or dissolving the coating of Cytodex 3 microbeads (F) result both in cell detachment while control cells (B and E) remain adherent. Data are expressed as means ± SEM.

**Figure 4:**
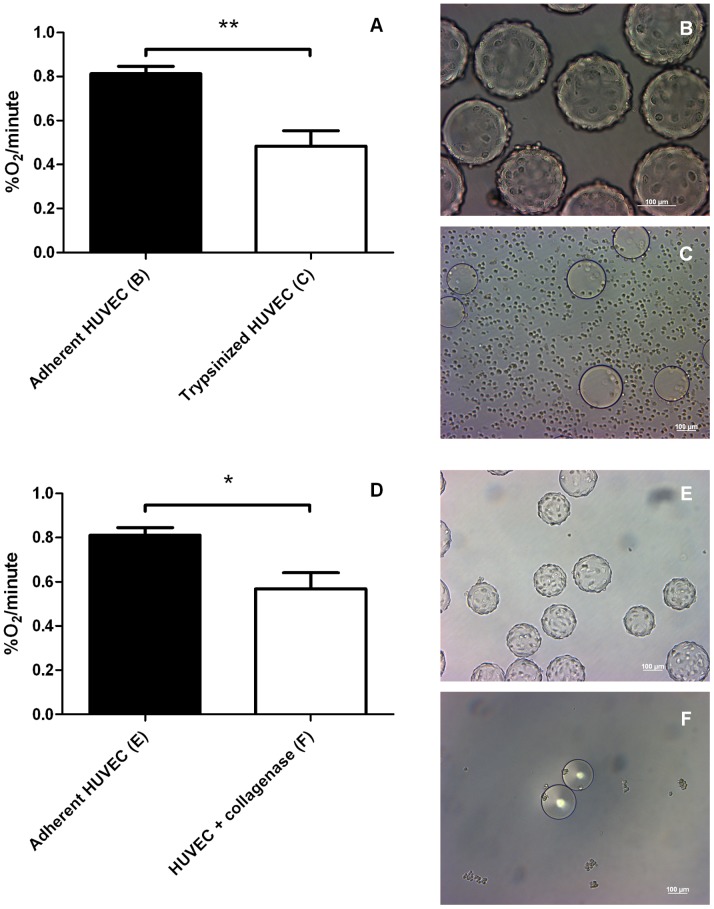
Effect of detachment procedures on HUVEC cells. OCR values (%O_2_/minute) (A, D) of adherent and detached HUVECs. Trypsinized (n = 3) or collagenase group (n = 3) show also a decreased oxygen consumption rate compared to adherent group (n = 3 for A and D). Results are statistically significant (**p<0.01 for trypsinized cells, *p<0.05 for HUVEC+collagenase). Microscopy pictures confirm cell detachment using trypsin (C) and collagenase (F) while control cells remain adherent (B, E). Data are expressed as means ± SEM.

Trypsinization of B16F10-luc murine melanoma cells induced a ∼60% decrease in the OCR (Figure 3A, 0.58±0.03%O_2_/minute for adherent *versus* 0.24±0.06%O_2_/minute for trypsinized cells). Trypsinization induced a similar although of less amplitude ∼40% reduction in the OCR of HUVECs (Figure 4A, 0.81±0.03%O_2_/minute for adherent *versus* 0.48±0.07%O_2_/minute for trypsinized cells). These data indicate that cell adhesion paces the oxidative metabolism of tumor and endothelial cells at a high rate, whereas cell detachment with trypsin induces a metabolic reprogramming towards a less oxidative phenotype. Cell survival was only moderately affected by the treatment, with a 94% B16F10-luc and a 91% HUVEC survival after trysinization.

**Figure 5:**
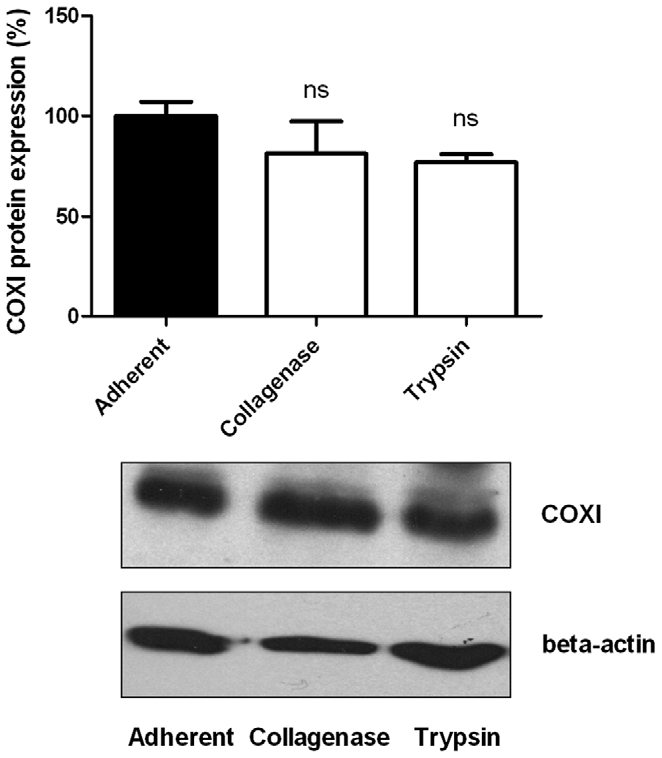
Effect of detachment procedures on COXI protein expression. Trypsinized cells (n = 3) or collagenase-treated cells (n = 3) have similar mitochondrial COXI protein levels than adherent cells (n = 3) (ns, *p*>0.05 (one way ANOVA) compared to adherent cells). Data are expressed as means ± SEM.

Since trypsinization is known to affect the expression of proteins that regulate cell growth, metabolism, adhesion,… [26], [27], we took advantage of collagen-coated cytodex 3 microbeads to use collagenase instead of trypsin to achieve cell detachment. The collagenase treatment of B16F10-luc-coated beads allowed efficient cell harvesting (Figure 3F). Also in these smoother experimental settings, cell detachment accounted for a net reduction in O_2_ consumption (Figure 3D, 0.75±0.08%O_2_/minute for adherent *versus* 0.49±0.09%O_2_/minute for detached cells). It was confirmed with HUVECs (Figure 4D, 0.81±0.03%O_2_/minute for adherent *versus* 0.57±0.07%O_2_/minute for the collagenase group). The collagenase treatment was found to be responsible for a less pronounced OCR inhibition (34% for B16F10-luc, 30% for HUVECs) compared to trypsin, while cell viability was totally preserved similarly to trypsin (data not shown). Our data collectively indicate that cell detachment generally reduces the OCR of tumor and endothelial cells. HUVECs were grown on Cytodex 3 and both harvesting methods were carried out from the same batch of cells, meaning that the same control was used for both treatments. Furthermore, to ensure that the observed decreases in the OCR reflect cellular stresses induced by detachment procedures and not experimental bias, mitochondrial COXI protein expression was assessed using Western Blotting (Figure 5). COXI expression was not significantly altered when cells were detached with trypsin or collagenase (100±7.02% COXI protein expression for attached cells, 81.06±16.23% for collagenase, 76.63±4.22% for trypsin).

### Cells in Suspension Undergo ATP Depletion, Altered Glucose Metabolism and Significant Cell Death

**Figure 6:**
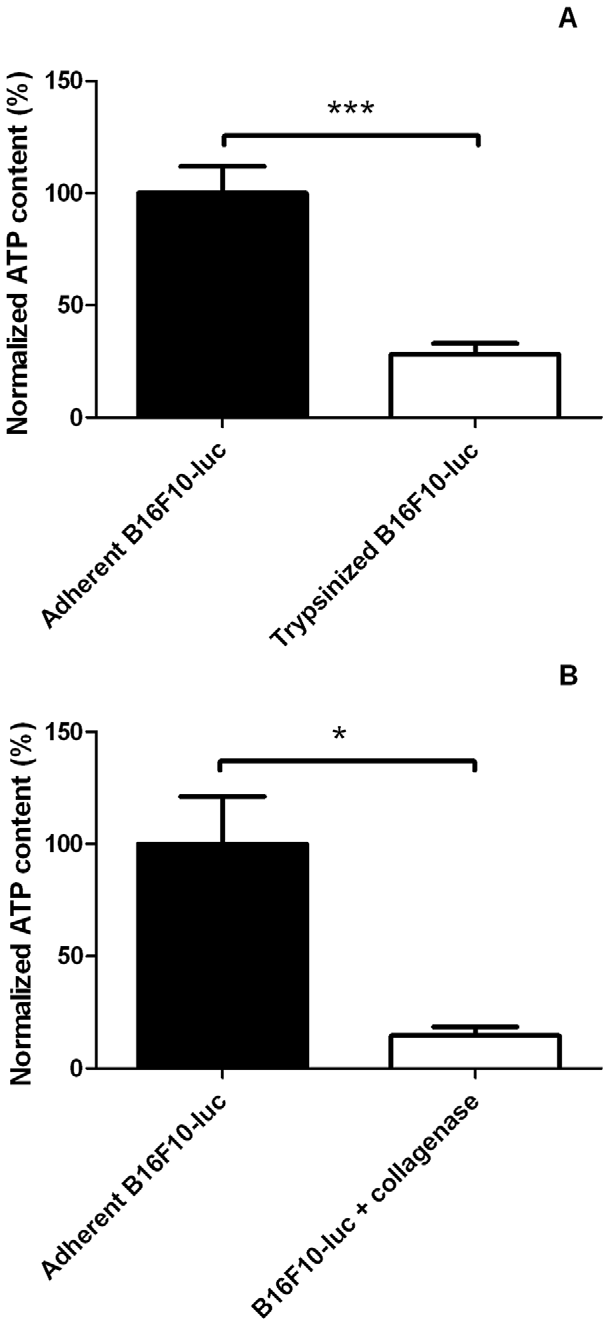
Influence of cell detachment on the intracellular ATP content of B16F10-luc. (A) Trypsinized cells (n = 6) display a marked ATP depletion compared to adherent cells (n = 6). (B) The same experiment was carried out using collagenase to harvest cells attached on collagen-coated plates (n = 3 for control and B16F10+ collagenase groups) and led to similar conclusions. Results are statistically significant (***p<0.001 for trypsinized cells, *p<0,05 for B16F10-luc+collagenase). Data are expressed as means ± SEM.

After having observed that cell detachment impairs mitochondrial respiration, we aimed to check whether keeping cells in suspension could affect their ATP content. As shown in Figure 6, intracellular ATP levels dropped 1 hour post detachment whatever the procedure. (Figure 6A, adherent B16F10-luc: 100.0±11.94% normalized ATP content, trypsinized B16F10-luc: 28.17±4.8% normalized ATP content; Figure 6B, adherent B16F10-luc: 100.0±21.13 normalized ATP content, B16F10-luc+collagenase: 14.64±3.87% normalized ATP content). Trypan blue viability tests performed 1 hour after detachment (trypsin and collagenase) did not show any significant cell death (data not shown).

**Figure 7:**
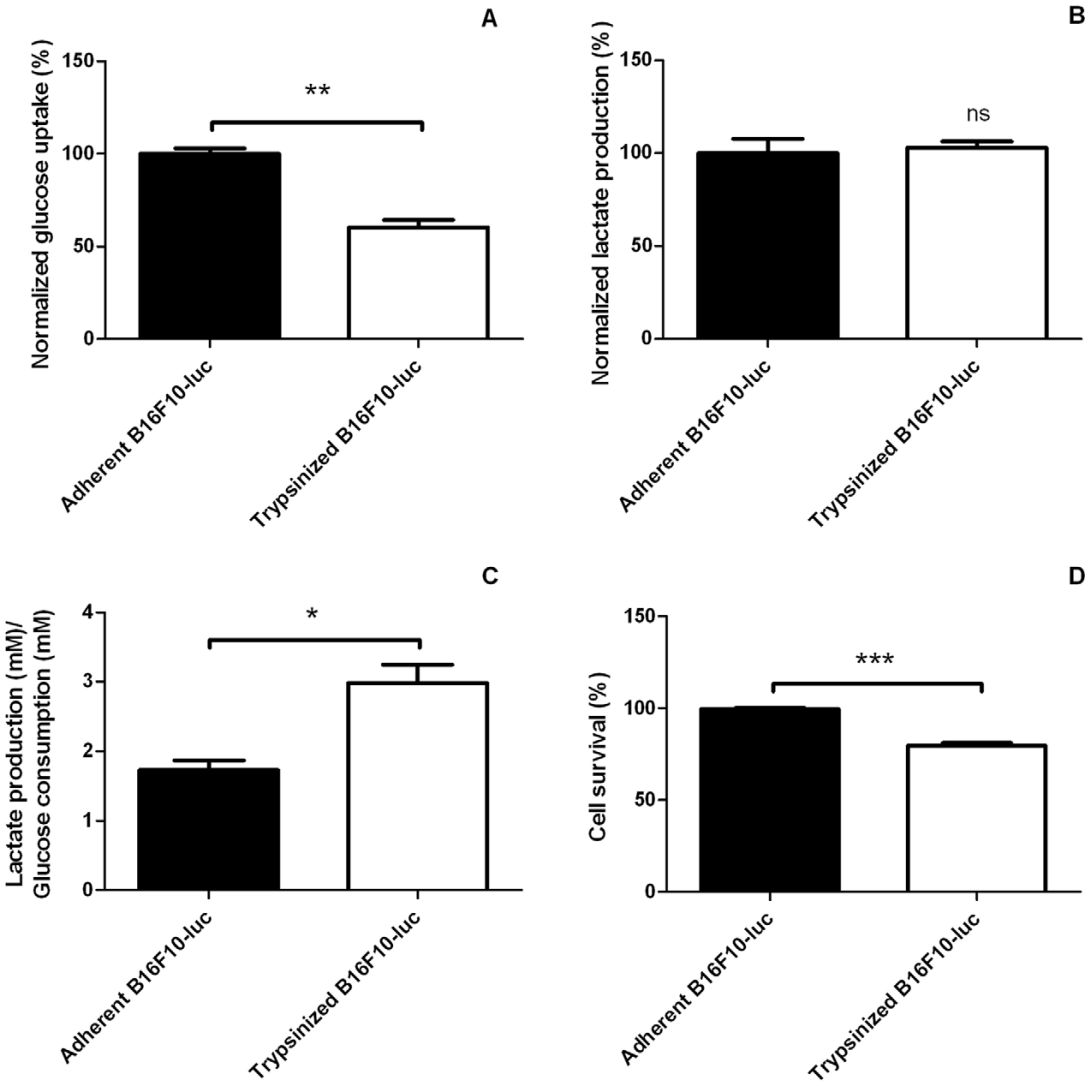
Glucose metabolism in adherent and trypsinized B16F10-luc. Trypsinized B16F10-luc (n = 3) take up less glucose (A) and release similar amounts of lactate (B) than adherent cells (n = 3). Cell detachment therefore accounts for an increased lactate production/glucose uptake ratio (C). Prolonged detachment (4 hours) affects cell survival (D). Results are statistically significant (**p<0.01 for A, ns, *p*>0.05 for B, *p<0.05 for C, ***p<0.001 for D). Data are expressed as means ± SEM.

**Figure 8:**
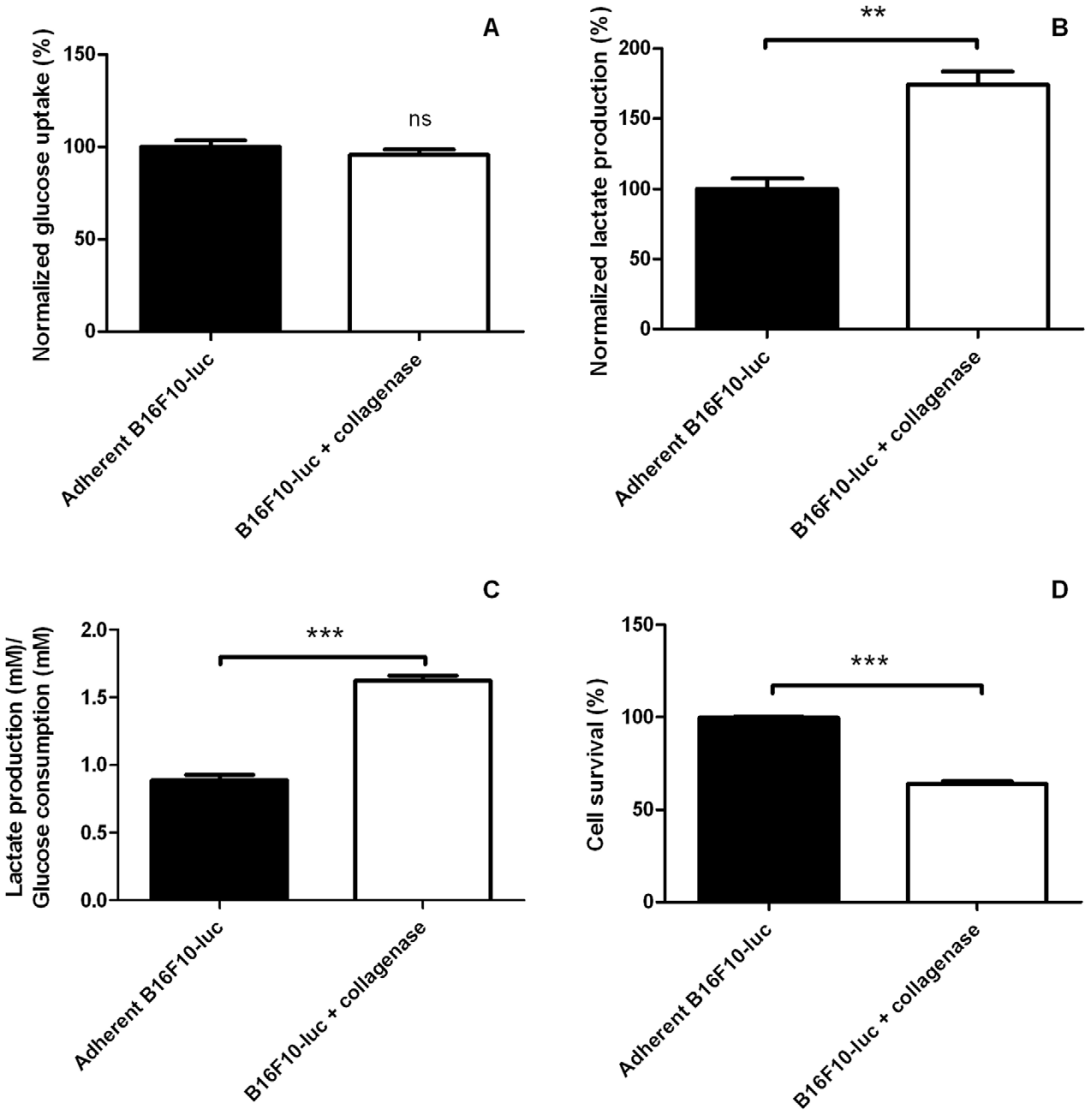
Glucose metabolism in adherent and B16F10-luc detached using collagenase. B16F10-luc seeded on collagen-coated plates and harvested using collagenase (n = 3) take up similar levels of glucose (A) but release more important amounts of lactate (B) than adherent cells (n = 3). Cell detachment results thus in an increased lactate production/glucose uptake ratio (C). Prolonged detachment (3 hours) affects cell survival (D). Results are sastistically significant (ns, *p*>0.05 for A, **p<0.01 for B, ***p<0.001 for C, ***p<0.001 for D). Data are expressed as means ± SEM.

Because mitochondrial activity was perturbated after a detachment procedure and because cells in suspension had lower amounts of intracellular ATP, we tested whether other main metabolic pathways were also perturbated. Glucose and lactate concentrations were measured after 3 hours (collagenase group) or 4 hours (trypsin group) after detachment. B16F10-luc in suspension after a trypsin treatment (Figure 7) consumed significantly less glucose (Figure 7A, adherent B16F10-luc: 100.0±3.03% normalized glucose uptake, trypsinized B16F10-luc: 60.38±4.01% normalized glucose uptake) but generated similar amounts of lactate compared with attached cells (Figure 7B, 100.0±7.73 and 103.0±3.30% normalized lactate production for adherent B16F10-luc and trypsinized cells respectively). The experiment was repeated using collagenase instead of trypsin (Figure 8). It showed in this instance that detached cells consumed similar levels of glucose (Figure 8A, 100.0±3.49% normalized glucose uptake for adherent group, 95.51±3.00% for detached group) but released more important amounts of lactate (Figure 8B, 100.0±7.46% *versus* 174.4±9.33% normalized lactate production for adherent B16F10-luc and B16F10-luc+collagenase respectively) compared with adherent cells. When considering the lactate production/glucose consumption ratio (glycolytic index), both harvesting methods led to an increased glycolytic index (Figure 7C for trypsin experiments, glycolytic index = 1.73±0.14 for adherent cells, 2.98±0.26 for trypsinized cells; Figure 8C for collagenase experiment, glycolytic index = 0.89±0.39 for adherent cells *versus* 1.625±0.36 for detached cells). Significant cell death was observed at later time points after cell detachment (Figure 8D, 63.91±1.38% survival in collagenase group; Figure 7D, 79.71±1.54% survival in trypsin group).

## Discussion

This *in vitro* study shows that detached cells consume highly significantly less oxygen than adherent cells, implying that cell adhesion promotes cell respiration and cell detachment protocols mitochondrial uncoupling. OCR inhibition appeared quickly after harvesting when viability was preserved. However, cells remaining in suspension had decreased intracellular ATP levels, which is in accordance with previously published results [28]. Although this net reduction in intracellular ATP is coherent with a decreased OCR, we cannot exclude that detached cells consume ATP much faster than adherent cells in order to maintain cellular homeostasis. We further observed that cells in suspension after both trypsin and collagenase treatments for a prolonged period (3–4 hours) exhibited a higher glycolytic index, indicating that other nutrients than glucose (such as glutamine which was present in the experimental medium) became a significant source of lactate when cells are detached. Eventually, a significant proportion of cells did not survive when kept in suspension for longer time. Surprisingly, survival was better for trypsin-treated cells compared to collagenase-treated cells. A reasonable explanation is that for this specific experiment, on the one hand trypsin exposure was much shorter and on the other hand vigorous pipetting was necessary to detach cells adherent to a collagen substrate when using collagenase. Altogether, we evidenced that detachment affects several key metabolic parameters.

Although other reports have already stated that mechanically detached cells or trypsinized cells have decreased metabolic activities (decreased glucose oxidation and oxygen consumption) [29], [30], our observations extend the paradigm to the less aggressive harvesting collagenase protocol and thereby show that numerous commonly used detachment methods may disturb mitochondrial respiration. Nevertheless, our study highlights that prolonged cell suspension is followed by important metabolic perturbations (glucose metabolism, intracellular ATP, …). There is therefore strong evidence that preserving cell adherence is a preferred condition for accurate metabolic studies.

Interestingly, for both B16F10-luc cells and HUVECs, OCR inhibition was more pronounced using trypsin than collagenase. Since trypsin acts through cleaving membrane proteins and collagenase is essentially a protease of the extracellular matrix, OCR inhibition upon detachment would involve not only the physical cell-surface and cell-cell interactions but also more generally signals originating from the cell membrane, involving anchor proteins or not, and influencing cell metabolism. Metabolic adaptations to cell detachment and loss of cell-matrix contacts have previously been studied in different setups. Accordingly, it is known that cells loosing contacts with their surrounding mainly *via* integrin signalization produce an increased level of reactive oxygen species (ROS), activate pro-apoptotic cascades, and eventually undergo a specific form of cell death termed anoikis [31], [32]. With a focus set on cell metabolism, it has been shown that detached cells have decreased glucose uptake rates affecting both glycolysis and the pentose phosphate pathway, and that oxidative stress following detachment impairs fatty acid oxidation [33]–[35]. Our observations are thus in line with these previous studies albeit using an innovative, sensitive and reproducible EPR approach allowing straightforward *in vitro* oxygen measurements.

Although the deregulation of major metabolic pathways is a pertinent cause of OCR inhibition, other mechanisms are probably involved. The existence of a transmembrane electron transport chain consuming oxygen to maintain the cellular redox status within a physiological range has been shown to be a significant contributor to global cell O_2_ consumption in addition to mitochondrial respiration [36]. Thus, altering the membrane organization with detachment might strongly affect these enzymatic processes. It could explain why trypsin, cleaving non specifically membrane enzymes, has a more important impact on OCR than collagenase for both studied cell lines (approximately 50% inhibition for trypsin and 30% for collagenase treatments). Cell detachment is also naturally followed by deep morphological changes. Harvested cells exhibit spherical shapes which differ from the adherent phenotype, implying that the actin cytoskeleton architecture and outside-in signaling would logically be altered following detachment procedures. Since many glycolytic enzymes are localized in the cytoplasm and sequestrated near actin filaments, their activities could be affected by such morphological alterations [37].

On a methodological point of view, the use of microcarrier technology and EPR oximetry combines the advantages of both techniques. Although oximetry methods on adherent cells have been described (such as with the Seahorse approach) [21], [38], [39] and cytodex beads have already been used in combination with Clark electrodes [40], our technology provides the unique advantage to be highly sensitive so that it can theoretically detect even small OCR changes likely due to biologically significant variations, whereas the stressful steps used here for proof-of-principle validation and with other techniques are relevant for sample preparation. Therefore, implementation of this new protocol could be a promising tool for further metabolic cell characterizations.
